# Vaccination of cattle with the *Babesia bovis* sexual-stage protein HAP2 abrogates parasite transmission by *Rhipicephalus microplus* ticks

**DOI:** 10.1038/s41541-023-00741-8

**Published:** 2023-09-27

**Authors:** Marta G. Silva, Reginaldo G. Bastos, Jacob M. Laughery, Heba F. Alzan, Vignesh A. Rathinasamy, Brian M. Cooke, Carlos E. Suarez

**Affiliations:** 1 Department of Veterinary Microbiology and Pathology, College of Veterinary Medicine, Washington State University, Pullman, WA USA; 2 Animal Disease Research Unit, United States Department of Agricultural - Agricultural Research Service, Pullman, WA USA; 3 Parasitology and Animal Diseases Department, National Research Center, Dokki, Giza Egypt; 4 Australian Institute of Tropical Health & Medicine, James Cook University, Cairns, QLD Australia

**Keywords:** Immunology, Parasitic infection

## Abstract

The apicomplexan parasite *Babesia bovis* is responsible for bovine babesiosis, a poorly controlled tick-borne disease of global impact. The widely conserved gametocyte protein HAPLESS2/GCS1 (HAP2) is uniquely expressed on the surface of *B. bovis* sexual stage parasites and is a candidate for transmission-blocking vaccines (TBV). Here, we tested whether vaccination of calves with recombinant HAP2 (rHAP2) interferes with the transmission of *B. bovis* by competent ticks. Calves vaccinated with rHAP2 (*n* = 3), but not control animals (*n* = 3) developed antibodies specific to the vaccine antigen. Vaccinated and control animals were infested with *Rhipicephalus microplus* larvae and subsequently infected with virulent blood stage *B. bovis* parasites by needle inoculation, with all animals developing clinical signs of acute babesiosis. Engorged female ticks fed on the infected calves were collected for oviposition, hatching, and obtention of larvae. Transmission feeding was then conducted using pools of larvae derived from ticks fed on rHAP2-vaccinated or control calves. Recipient calves (*n* = 3) exposed to larvae derived from control animals, but none of the recipient calves (*n* = 3) challenged with larvae from ticks fed on rHAP2-vaccinated animals, developed signs of acute babesiosis within 11 days after tick infestation. Antibodies against *B. bovis* antigens and parasite DNA were found in all control recipient animals, but not in any of the calves exposed to larvae derived from HAP2-vaccinated animals, consistent with the absence of *B. bovis* infection via tick transmission. Overall, our results are consistent with the abrogation of parasite tick transmission in rHAP2-vaccinated calves, confirming this antigen as a prime TBV candidate against *B. bovis*.

## Introduction

Bovine babesiosis caused by the tick-borne apicomplexan parasite *Babesia bovis* is an economically important disease that threatens the cattle industry in the US and worldwide^1,2^. The disease results in high mortality and morbidity and is characterized by anemia, fever, and sequestration of parasitized red blood cells (RBC) in blood capillaries, a feature shared with the related arthropod-borne apicomplexan parasite *Plasmodium falciparum*, that leads to the development of neurological symptoms^3,4^.

Common strategies for the control of bovine babesiosis include the use of acaricides, anti-babesial drugs, and live attenuated vaccines^1,5^. Eradication of tick vectors using acaricides is conceptually the most efficient method for preventing bovine babesiosis but is unpractical, may have high toxicity, and cause detrimental environmental effects. More worrisomely, continuous use of acaricides consistently leads to the development of resistance by the ticks^6^. In addition, anti-babesia drugs are costly, may interfere with the development of herd immunity against the parasite, and may also generate toxic metabolic residues in the food chain, resistance, and toxicity^7^. Live vaccines based on blood-stage attenuated parasites are relatively effective but can only be administrated to less than one-year-old calves and may be transmissible by ticks. In addition, live vaccines pose the risks of contamination with other pathogens and potential reversion to virulence^2,8^. Therefore, the currently available strategies to control bovine babesiosis are limited, and new approaches are urgently needed. An ideal alternative to live vaccines is the use of subunit vaccines able to prevent acute babesiosis. Such recombinant subunit vaccines could be composed of a single or a combination of several protective antigens expressed in blood stages of *B. bovis* and formulated in a suitable adjuvant. Several candidate antigens have been identified and tested as possible components for subunit vaccines, but none have succeeded in providing sufficient levels of protective immunity, and thus subunit vaccines so far remain unavailable^1,5,9^. Subunit vaccine trial failures have been attributed in part, to the complexity of the parasite–host relationship, including the parasite’s ability to escape the host’s immune responses using different mechanisms, such as antigenic variation, capillary sequestration, and redundant pathways for RBC invasion. In addition, the lack of well-defined correlates of protection also limits the rational design of subunit vaccines^10^. In view of the grand difficulties encountered in the development of vaccines aimed at controlling the devastating effects of acute babesiosis, blocking the transmission of the parasites in tick vectors emerges as an attractive strategy that so far remains unexplored to limit the impact of the disease. This approach is particularly promising because, in contrast to blood-stage antigens, specific tick-stage parasite antigens are not likely subjected to pressures from the vertebrate host immune selection that invariably drives the emergence of antigenic variants in parasite populations. Therefore, *B. bovis*-specific proteins that are surface expressed in the sexual stages of the parasite developing in the midgut of their definitive tick hosts, are rational targets for transmission-blocking vaccines (TBV). While we continue pursuing the identification of effective candidates and adequate formulations for developing an effective blood-stage vaccine against *B. bovis*, clearly, the development of TBV could vertically advance the control of bovine babesiosis. Ideally, TBV candidates could be combined with effective blood-stage antigens to generate a dual blood-stage/transmission-blocking subunit vaccine that may present a viable alternative to current live vaccines, which are not known to be able to generate immune responses that limit transmission of the parasite by ticks.

The lifecycle of *B. bovis* is complex, including sexual reproduction in the midgut of its biological tick vectors, mainly *Rhipicephalus microplus* and *R. annulatus*, and asexual reproduction inside cattle erythrocytes^11,12^. Importantly, these two are monoxenic species of ticks, therefore they develop their full lifecycle, consisting of larval, nymphal, and adult stages, on a single host. The mechanism of sexual reproduction of *B. bovis* has not been fully elucidated at the molecular level, but previous studies identified key proteins involved in this complex process, including the gene expression regulators AP2 proteins^13^, the widely conserved 6cys and CCp proteins^13–15^, and the HAPLESS 2 (HAP2) protein^16^. The first *B. bovis* transmission-blocking vaccine trial performed in cattle was based on the 6cys A and 6cys B proteins. Although vaccination of cattle with these two tick-stage specific proteins failed in eliciting protective immune responses against transmission of the parasite, it provided valuable lessons on their immunogenicity and important insights for the design of vaccine trials for TBV against *B. bovis*^17^.

HAP2 is a functionally conserved membrane-associated protein, homologous to class II viral fusogen, expressed by male gametocytes, and essential for gamete fusion in a diverse array of eukaryotes, including plants, protozoan, and other metazoans^18–21^. Expression of HAP2, a protein that was originally identified in *Arabidopsis thaliana*, is a hallmark of sexual reproduction in several eukaryotic organisms^22^. In fact, it was demonstrated that HAP2 is essential for zygote formation and therefore, critical for successful sexual reproduction. HAP2 is well-conserved among apicomplexans and in multiple eukaryotic organisms. The single copy *hap2* gene of *B. bovis* has been recently shown to be differentially expressed by parasites in the tick midgut and on the surface of in vitro-induced sexual stages of the parasite^16^. Consistently, the *hap2* gene was also found expressed in midgut stages of the highly related *B. bigemina*^23^, where it may also play a key role in the development of sexual stages and fertilization in this parasite. Expression of HAP2 has also been recognized as critical for the fertilization of the *Babesia*-related *Plasmodium* parasites^24,25^. Importantly, both *Babesia* and *Plasmodium* are apicomplexan parasites that share the occurrence of sexual reproduction in the midgut of *Rhipicephalus* ticks and *Anopheles* mosquitoes, respectively. Additionally, immunization with recombinant HAP2 of *P. falciparum* induced transmission-blocking antibodies^25^. Also, it was shown that specific antibodies against HAP2 of *B. bigemina* are able to block zygote formation^23^. In addition, we previously demonstrated that the knockout of the *B. bovis hap2* gene impairs the development of sexual stages in vitro^16^, demonstrating that HAP2 might play an essential role in the mechanism of sexual replication of the parasite. Recent experiments using an artificial feeding system for *Anopheles dirus* demonstrated the ability of anti-HAP2 antibodies to interfere with the sexual reproduction of *P. vivax*^26^. Collectively, this body of work provides a strong rationale supporting HAP2 as a candidate for the development of TBV against arthropod-transmitted *Babesia* and *Plasmodium* apicomplexan parasites. However, all these previously published studies were performed using in vitro models of tick feeding or sexual stage induction, and, at least to our knowledge, no vaccine trials of HAP2 involving natural hosts and acquisition-transmission models have ever been reported.

We hypothesize that the induction of antibodies against *B. bovis* HAP2 in bovines via vaccination with a recombinant version of the protein (rHAP2) will interfere with the transmission of the parasite by competent tick vectors. Thus, the aim of this study was to investigate the suitability of rHAP2 as a TBV against *B. bovis* using a bovine/competent tick model. Our results demonstrate that vaccination with rHAP2 abrogates transmission of *B. bovis* by *R. microplus* ticks, a pivotal observation that sets the foundation for the development of a TBV to protect against infection with *B. bovis* and bovine babesiosis.

## Results

### Vaccination of calves with rHAP2 followed by challenge with *R. microplus* larva and virulent *B. bovis*

**Fig. 1:**
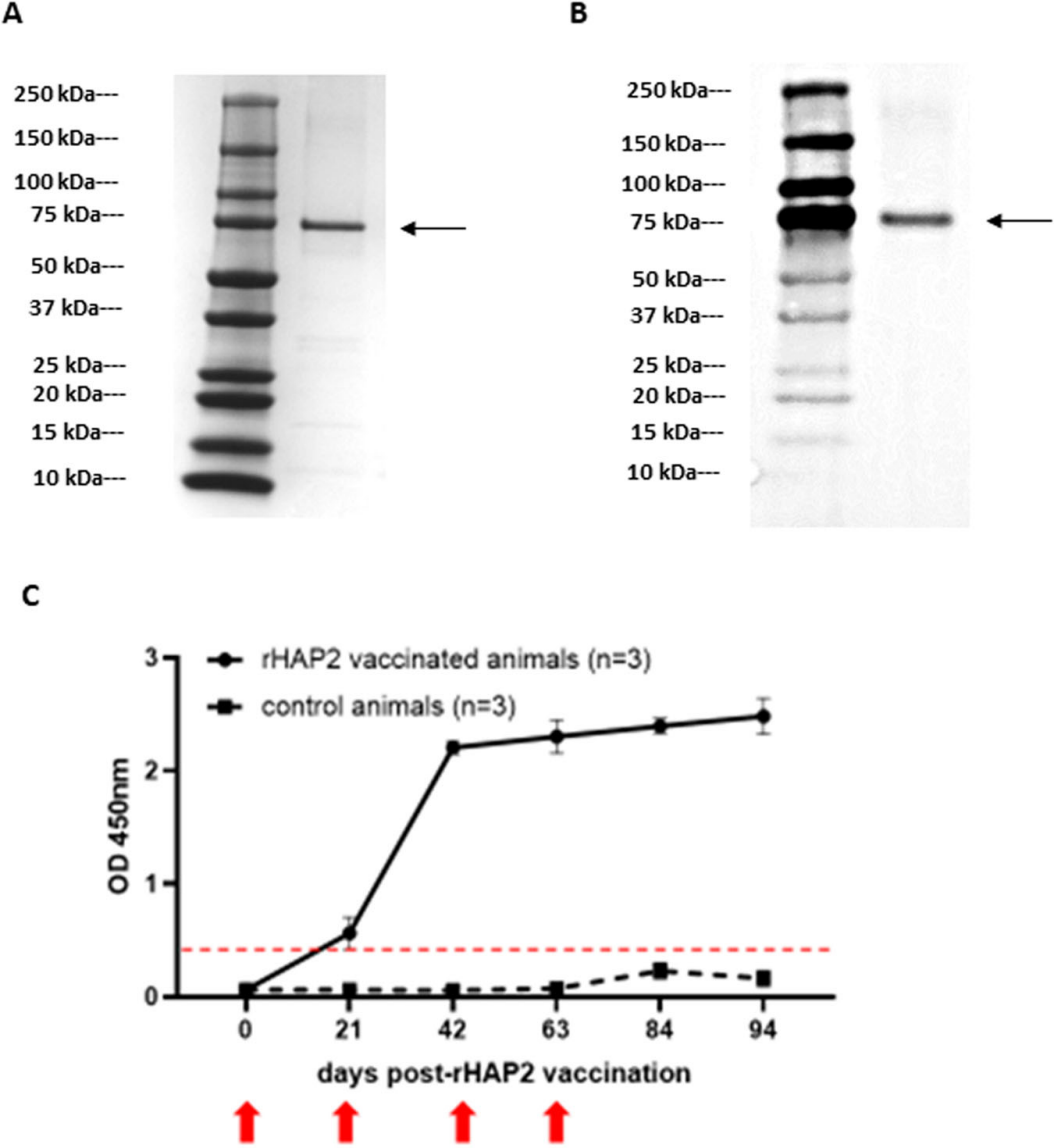
Cattle immunization. SDS–PAGE (**A**) and immunoblot (**B**) analysis of Ni-column purified *B. bovis* rHAP2 using mouse anti-6His antibody. The rHAP-2 protein is identified by arrows. The size of protein markers is indicated on the left. **C** Serological analysis by iELISA for the detection of anti-rHAP2 antibodies on rHAP2 vaccinated calves (1646, 1647, and 1649), and non-vaccinated controls (1650, 1652, and 1653). The *Y* and *X* axes represent absorbance at 450 nm and days after vaccination (days 0–94), respectively. The red dashed line represents the cutoff value of the iELISA, calculated as the OD of non-infected cattle sera +3 standard deviations. The error bars represent the standard deviations of samples at each time point.

**Fig. 2:**
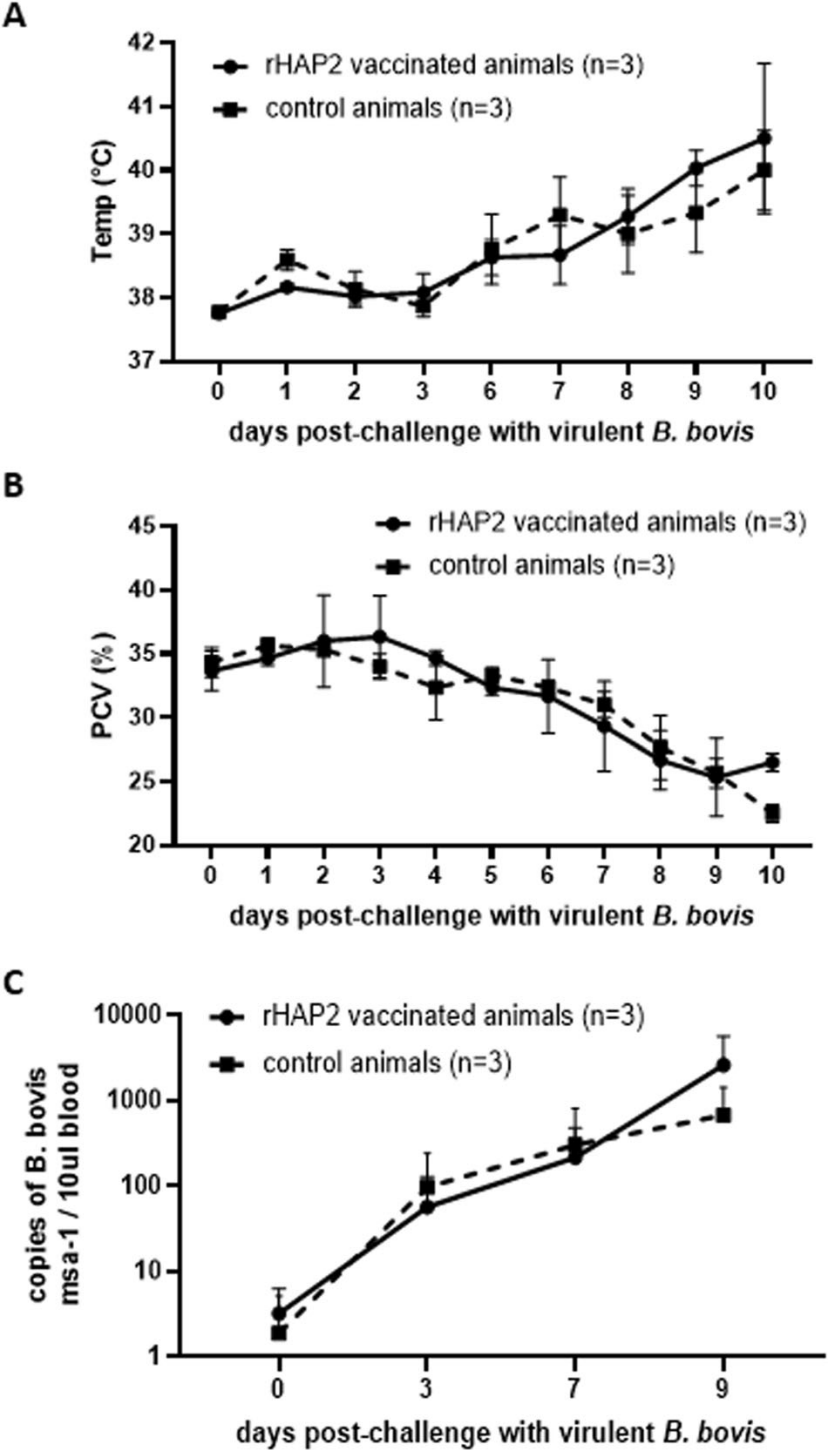
Measurement of clinical parameters on acquisition feeding calves challenged with *B. bovis* S74-T3Bo Texas strain. **A** Rectal temperature in ^o^C is shown on the *Y*-axis and the days post challenge on the *X*-axis. **B** Packed cell volume (PCV) is shown on the *Y*-axis, and the days post-challenge are presented on the *X*-axis. **C** Quantification of the average number plus standard deviation of copies of *B. bovis msa-1* gene by qPCR on gDNA extracted from the blood of infected calves at days 0−, 3−, 7−, and 9-day post-infection. The error bars represent the standard deviations of samples at each time point.

HAP2 is a sexual stage protein of *B. bovis*, exclusively expressed in the midgut of the tick vector^14^. However, whether cattle infected with *B. bovis* generate antibodies against HAP2 was not previously reported. Therefore, we used rHAP2 and rRAP-1 indirect ELISAs (iELISA) to compare the levels of anti-HAP2 and RAP-1 antibodies in sera from eight *B. bovis-vaccinated* cattle that were also challenged with a virulent *B. bovis* strain. While sera from all vaccinated and challenged cattle contain antibodies that reacted with RAP-1, an immunodominant blood stage antigen of *B. bovis*, none of them showed antibodies to rHAP2 in the assays (Supplementary Fig. 1). Next, we first investigated the immunogenicity of *B. bovis* rHAP2 in cattle by vaccinating three calves (c1646, c1647, and c1649) with rHAP2, while three control calves (c1650, c1652, and c1653) were inoculated with adjuvant alone (Fig. 1A and B). Serological analysis using the rHAP2-iELISA demonstrated that all three vaccinated calves developed significant levels of antibodies against rHAP2, while no such reactivity was detected using sera from control animals (Fig. 1C). A significant increase in the levels of antibody response against rHAP2 was detected at day 42 post-inoculation (PI) after the second boost inoculation with rHAP2. Similar levels of anti-HAP2 antibodies were maintained throughout the experiment until day 94 PI. All experimental calves were then challenged sequentially with *R. microplus* larvae and a virulent, tick transmissible, strain of *B. bovis*. Thus, all six experimental calves were first exposed to *R. microplus* larvae on day 72 PI with rHAP2. This was then followed by a challenge with a blood stabilate containing 10^7^ *B. bovis* S74-T3Bo Texas strain infected erythrocytes, using the IV route at day 84 PI (12 days after tick infestation). The timing of larval application was chosen to synchronize the dropping of fully engorged adult female ticks with the expected peak of infection with *B. bovis*, favoring parasite acquisition by the ticks^13^. Starting at 7 days post *B. bovis* S74-T3Bo infection, the mean rectal temperature increased in a similar fashion in both vaccinated and control calves (Fig. 2A). In addition, packed cell volume (PCV) started decreasing 5 days after *B. bovis* challenge in all six experimentally infected calves (Fig. 2B). The timing and intensity of alterations in temperature and PCV occurring in all challenged calves are consistent with typical clinical markers of acute bovine babesiosis and fully consistent with previous similar experiments in our lab involving acute infection of cattle with the *B. bovis* S74-T3Bo Texas strain^17^. Overall, no significant differences were found among rHAP2-vaccinated and control groups regarding changes in body temperature (*P* = 0.97) and PCV (*P* = 0.98) during acute infection. Additionally, all experimentally infected calves showed other typical clinical signs of acute babesiosis, such as loss of appetite and apathy, regardless of their vaccination status.

We also evaluated the amounts of parasite DNA circulating in peripheral blood by quantitative PCR (qPCR) analysis performed on total DNA extracted from the animals at different times during the experimental infection. The single copy *B. bovis msa-1* gene was used as a target to detect parasite DNA by qPCR. Parasite DNA was found in all infected calves at variable levels among all the time points tested (Fig. 2C). However, no statistical differences were found in parasite load by using a non-parametric test on 3 (*P* = 0.99), 7 (*P* = 0.99), and 9 (*P* = 0.70) days post-*B. bovis* infection by comparing rHAP2-vaccinated and control calves. Taken together, these data demonstrate that vaccination with rHAP2 elicited a strong antibody response in the experimental calves, but as expected, this immunization had no effect on the natural clinical course of acute infection upon IV challenge with the *B. bovis* virulent strain S74-T3Bo. The majority of engorged *R. microplus* female ticks dropped after 9 days post-*B. bovis* infection in all experimental animals. Eggs laid by the engorged female ticks were used for transmission-feeding experiments described below.

### Reproductive fitness in ticks fed on rHAP2 vaccinated calves

**Fig. 3:**
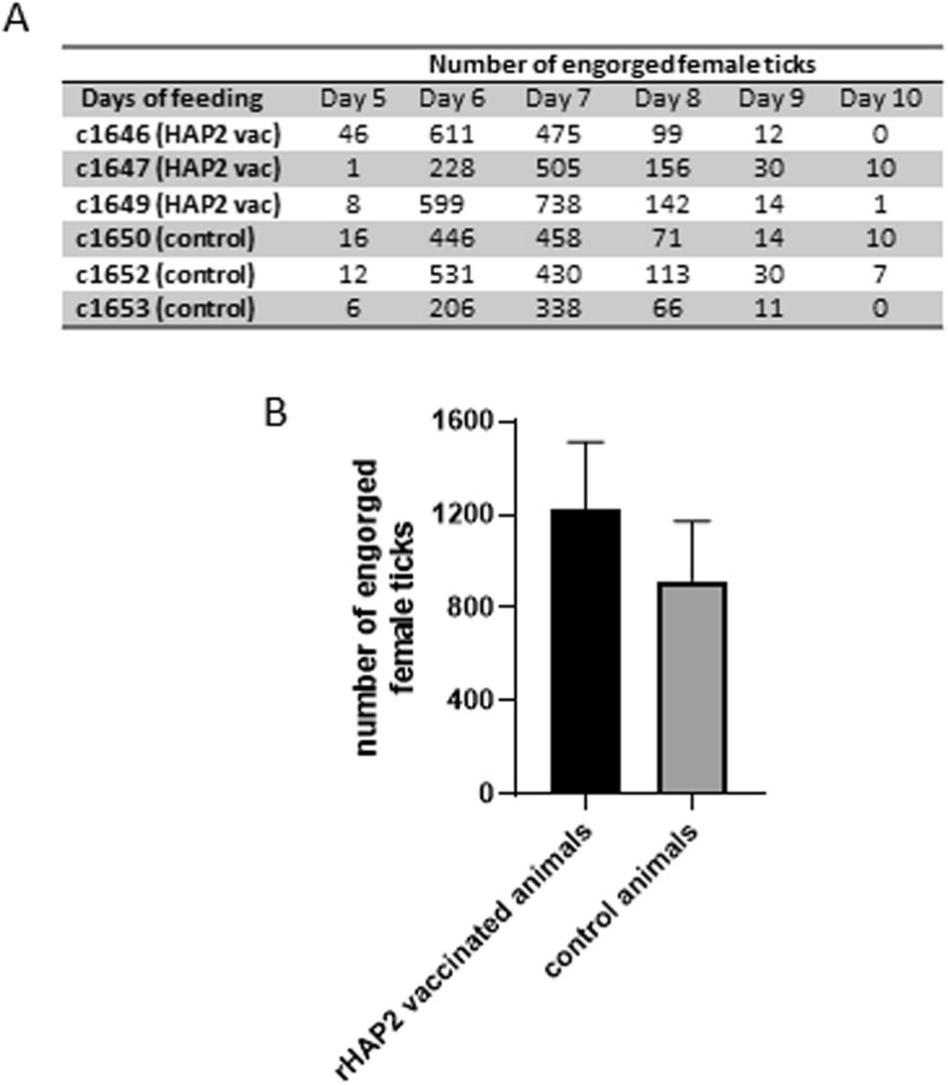
Analysis of fitness of ticks feeding on vaccinated (1646, 1647, and 1649) and control (1650, 1652, 1653) calves at days 5–10 after *B. bovis* infection. **A** Number of engorged female *R. microplus* ticks recovered from rHAP-2-vaccinated and control. **B** Total number of engorged female *R. microplus* ticks recovered from rHAP-2-vaccinated and control. The error bars represent standard deviations of the number of engorged female ticks in vaccinated and control animals.

It is conceivable that *B. bovis* infection in ticks may affect tick fitness. Considering our hypothesis that immune responses of rHAP2-vaccinated cattle interfere with the lifecycle of the parasite within competent ticks, it was of interest to investigate whether there were differences in fitness among ticks feeding in vaccinated or non-vaccinated control calves. To this end, we evaluated first the numbers of replete female ticks recovered, total egg masses, and the number of *B. bovis* kinetes present in hemolymph in randomly selected replete ticks (72 ticks per experimental animal) fed on vaccinated and non-vaccinated calves. The total number of replete ticks recovered per animal is shown in Fig. 3A. We also determined the average numbers of engorged ticks that dropped from both groups of cattle (Fig. 3B). Statistical analysis showed no significant difference among the two groups, despite a tendency of more ticks dropping from the vaccinated group.

The total weight of ticks from the vaccinated animals was 207.9 g (*n* = 759), while the total weight of ticks obtained from the control animals was 184.4 g (*n* = 728). Despite this trend, no significant difference was found between the vaccinated and control groups (*P* = 0.06).

The presence of kinetes in 72 replete female ticks per calf (vaccinated and control) from 5 to 9 days post-inoculation (dpi) of *B. bovis* was evaluated by microscopic analysis. Kinetes were observed only in the hemolymph of two ticks 8 dpi, which were derived from the control animals C1650 and C1652, respectively.

In addition, the weight of egg mass from ticks fed on vaccinated and control groups was statistically different (*P* = 0.023) (Table 1). The total egg weight of the initial 5 days of oviposition was 57.9 g for ticks developed in vaccinated animals (*n* = 556) and 49.4 g for ticks derived from control animals (*n* = 499). Thus, taken together, the data suggest that the development of *B. bovis* sexual stages inside the tick midgut, which presumably only occurred in ticks that fed on control animals, might affect the reproductive fitness of the ticks.

**Table 1:** Weight of egg mass derived from non-vaccinated and vaccinated calves.

	Weight of egg mass (g)
Non-vaccinated	49.4 (*n* = 499)
Vaccinated	57.9* (*n* = 556)
Mean	53.65

*Denotes statistical significance (*p* < 0.05).

### Vaccination with rHAP2 abrogates transmission of *B. bovis* by *R. microplus*

**Fig. 4:**
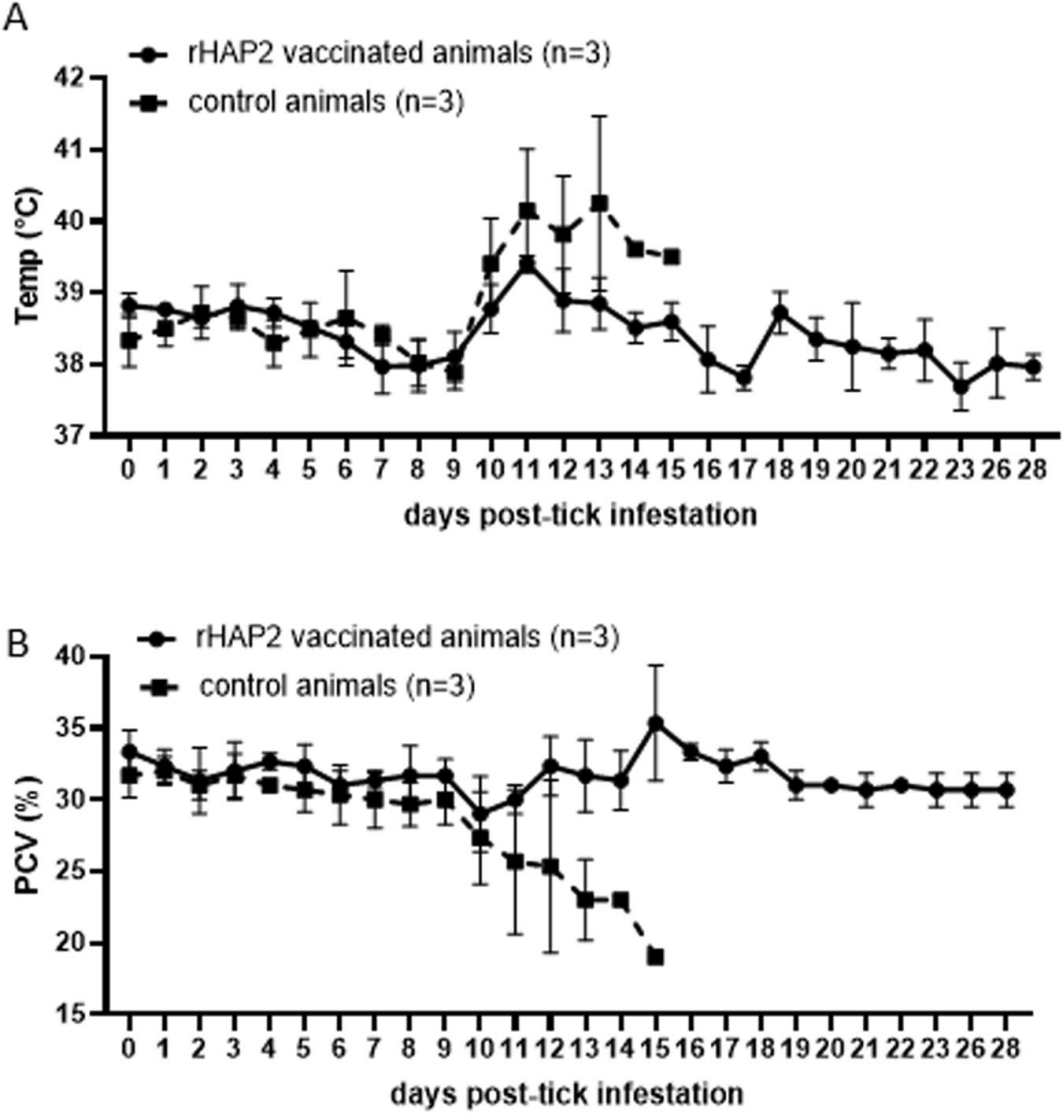
Measurement of clinical parameters on transmission feeding calves challenged with tick larvae derived from vaccinated (1646, 1647, and 1649) or control calves (1650, 1652, and 1653) infected with *B. bovis* S74-T3Bo Texas strain. **A** Rectal temperature in °C is shown on the *Y*-axis and the days post challenge on the *X*-axis. **B** Packed cell volume (PCV) on the *Y*-axis, and the days post challenge on the *X*-axis. Calves C99555; C99556, and C99557 received larvae derived from vaccinated calves, and C99559, C99561, and C99562 received larvae derived from control calves. The error bars represent the standard deviations of samples at each time point.

**Fig. 5:**
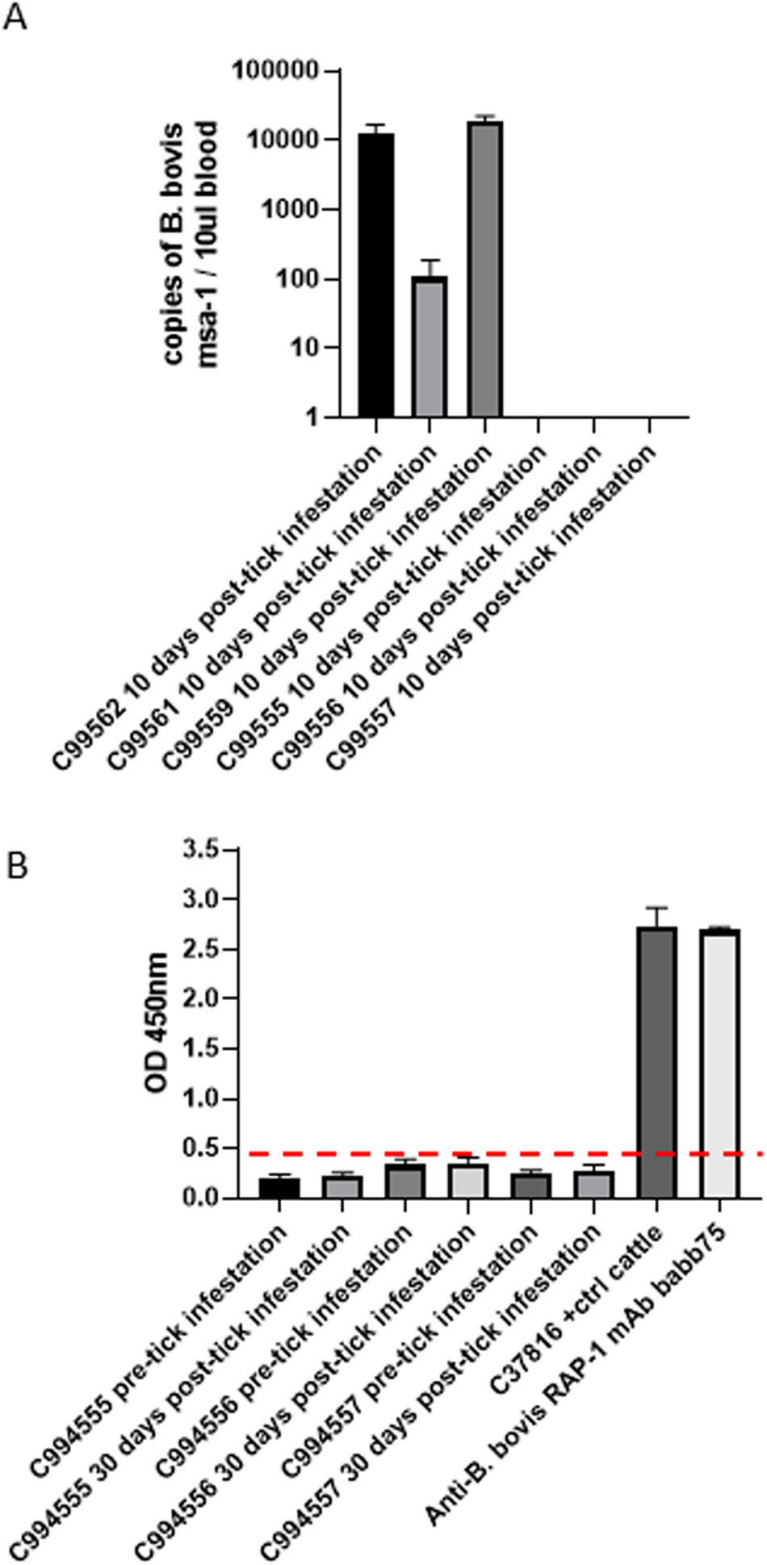
Assessment of *B. bovis* transmission by *R. microplus* larvae derived from vaccinated and non-vaccinated cattle. **A** Detection of *B. bovis* DNA by quantitative PCR (qPCR) based on the *B. bovis msa-1* gene on DNA extracted from blood from calves receiving ticks derived from vaccinated (C99555; C99556, C99557), or non-vaccinated control (C99559, C99561, C99562) calves. The *Y*-axis represents the gene number copies detected. The *X*-axis represents the timing for the extraction of each blood sample (days post-tick infestation of the cattle). **B** Detection of antibodies against the *B. bovis* RAP-1 protein by iELISA on sera from calves receiving ticks derived from vaccinated (C99555; C99556, C99557), or non-vaccinated control (C99559, C99561, C99562) calves. C37816 represents iELISA analysis performed sera from a calf experimentally infected with *B. bovis*. BABB75 represents iELISA performed with a monoclonal antibody reactive with RAP-1. The *Y*-axis represents OD at 435 nm. The red dashed line represents the cutoff value of the iELISA, calculated as the OD of non-infected cattle sera +3SD). The error bars in Panel **A** represent standard deviations of the number of msa-1 copies in experimental animals. The error bars in Panel **B** represent standard deviations of the OD 450 nm values in experimental animals.

The natural cycle of transmission of *B. bovis* includes the acquisition of infected erythrocytes by adult *Rhipicephalus* ticks feeding on infected bovines, followed by sexual reproduction in the tick midgut, and transovarial transmission to offspring larvae. Then, the infected larvae can transmit the parasite to naïve cattle in a new cycle of feeding. Therefore, it was of interest to investigate the ability of larvae from ticks fed on rHAP2-vaccinated animals to transmit the parasite to naïve calves. To this end, we evaluated the development of acute signs of *B. bovis* infection in two groups of three naïve calves that received either pooled *R. microplus* larvae from ticks collected from vaccinated calves or larvae from ticks fed on control animals. Clinical parameters were compared among all experimental calves. None of the calves infested with larvae from vaccinated animals displayed detectable fever or a significant decrease in PCV (Fig. 4A and B). In contrast, the three control calves that were infested with offspring larvae from ticks fed on the control unvaccinated group showed fever (>38.8 °C), a significant drop in PCVs (30%), and typical clinical symptoms of acute babesiosis, such as prostration, anorexia, and increased respiratory lethargy (Fig. 4A and B). All experimental calves were also analyzed for the presence of parasite DNA by qPCR and for the induction of humoral immune responses against *B. bovis* by iELISA. The presence of *B. bovis* DNA was detected in all control animals, but not in the three calves that received tick larvae from the HAP2-vaccinated animals (Fig. 5A). In addition, none of these three asymptomatic animals developed antibodies against *B. bovis* by the end of the experiment, 30 days after exposure to the ticks (Fig. 5B). Control calves were humanely euthanized at day 13 after larval infestation due to the severity of acute babesiosis. Collectively, results demonstrated that vaccination with rHAP2 abrogates the transmission of *B. bovis* by larvae of *R. microplus* ticks.

## Discussion

*Babesia* parasites have a complex lifecycle that includes sexual reproduction inside the midgut of tick vectors and asexual reproduction in their vertebrate hosts^12^. Sexual reproduction is a well-coordinated process that culminates in the formation of male and female gametes that fuse to form zygotes inside the midgut of ticks. Therefore, this mode of reproduction is an essential event in the lifecycle of *B. bovis* that assures its transovarial transmission by competent ticks, generates genetic diversity, facilitates further propagation of the parasites, and can also be an effective target for novel strategies for controlling this parasite. The molecular processes involved in *Babesia* sexual reproduction remain largely uncharacterized; however, it is likely that unique tick midgut factors, such as temperature, pH, and metabolites, play a role in triggering changes in the incoming blood stage parasites, in preparation for the events leading to sexual reproduction. Transformation of the blood-stage parasite into gametes involves dramatic phenotypical changes that require the differential expression of stage-specific genes, such as members of the AP-2 family, the 6cys, and CCp gene families, and HAP2^13–15,17,27^. The HAP2 protein was shown to be expressed on the surface of *Plasmodium* male gametes^28^, and it is likely involved in gamete fusion^29^. Consistent with these observations, the *B. bovis* HAP2 was also shown expressed on the surface of in vitro induced sexual stage parasites^16^. In addition, the HAP2 protein appears not to be expressed in blood-stage parasites^16^, and in contrast with *P. falciparum*^26^, we did not find antibodies against HAP2 in cattle experimentally infected with *B. bovis*. Considering the surface expression of HAP2, we predict that this sexual stage protein is exposed to molecules present in the tick midgut milieu, including antibodies and immune effector cells of the mammalian host. Altogether, these findings strongly support the use of HAP2 as a candidate for developing TBV against *Babesia* and other apicomplexan parasites.

Based on this evidence, we tested rHAP2 as a TBV against *B. bovis* using a tick-transmission model based on cattle, the natural host for the parasite. Strikingly, the results demonstrate the ability of rHAP2 to elicit an immune response in vaccinated cattle that fully abrogates transmission of *B. bovis* by *R. microplus*. Therefore, the results are in full support of the hypothesis that immune responses against *B. bovis* HAP2 in vaccinated bovines block the transmission of the parasite by a competent tick vector. As expected for an antigen uniquely expressed during sexual stages, rHAP2 vaccination had no effect on the onset and development of acute babesiosis in vaccinated cattle.

Transmission-feeding data showed the absence of acute babesiosis symptoms in recipient calves infested with larvae from ticks fed on rHAP2-vaccinated animals. Consistent with this observation, no detectable *B. bovis* DNA and seroconversion was observed in the recipient animals, suggesting that larvae from ticks fed on vaccinated calves, in contrast to those derived from ticks fed on control animals, were unable to transmit the parasite to naïve calves. Abrogation of parasite transmission suggests that immune responses against HAP2, likely soluble factors, such as antibodies and components of the complement system, were able to successfully block sexual reproduction of the parasite in the midgut of ticks. The most likely explanation for this observation is that HAP2 vaccination elicited antibodies that effectively bind surface-exposed HAP2 and prevent gamete membrane fusion during the fertilization process^24^. It is also possible that antibodies against HAP2 may prevent male–female gamete interactions by spatial hindrance. The absence of *B. bovis* kinetes in the hemolymph of ticks fed on vaccinated animals supports a blockage of the process of zygote formation in the midgut and the inability of the parasite to subsequently invade ovaries and accomplish transovarial transmission.

These results corroborate previous observations showing that HAP2 immunization, more specifically vaccination with its amphipathic domains, completely blocked *P. falciparum* and *P. berghei* transmission in the mouse model^24^. Vaccination with HAP2 induced significant production of antibodies, indicating that, despite its absence of expression in blood stages, this parasite sexual stage protein is immunogenic for cattle. Similar results of immunogenicity of the *Plasmodium* HAP2 were described previously in the mouse model^24^.

Considering that anti-HAP2 antibodies, present in the blood of vaccinated animals are taken up by ticks during a blood meal, it is tempting to speculate on the effect of these factors on parasite load inside ticks and their possible impact on tick fitness. Interestingly, we only found a decrease in fitness among ticks derived from non-vaccinated calves, consistent with the lack of *B. bovis* infection in ticks feeding in vaccinated calves. Taking these data together and considering that both HAP2-vaccinated and control animals were acutely infected with *B. bovis*, it is possible to infer the potential detrimental effect of the parasite infection on tick reproductive fitness, which is not evident in the ticks fed on vaccinated calves. Therefore, it is conceivable that the observed reduction in the reproductive fitness of ticks fed on control calves may be due to the presence of *B. bovis* parasites circulating in the ticks, which were apparently absent in the ticks fed on vaccinated animals. It has been previously shown that *B. bovis* infection can decrease the fitness of *R. microplus* and that the severity of the effect is associated with the level of parasitemia and tick strain susceptibility^30^. In fact, it was demonstrated that *B. bovis* infection changes the protein expression profile of *R. microplus* female ticks^31,32^ Thus, our results reinforce previously published data and show that the level of *B. bovis* infection may negatively affect the fitness of *R. microplus*.

In summary, several lines of evidence in this study demonstrate that vaccination of cattle with rHAP2 abrogates the development of sexual reproduction of *B. bovis* in its natural definitive tick host. Consistently, ticks fed on vaccinated calves were unable to transmit the parasite to naïve calves. Thus, vaccination of cattle, the natural vertebrate host of *B. bovis*, with HAP2 elicited immune responses that resulted in the blockage of the natural cycle of the parasite in a competent tick vector. Collectively, these results define HAP2 as a rational candidate antigen for the development of a TBV against *B. bovis*.

Future work will be aimed at determining whether vaccination with HAP2 is able to prevent transmission of different strains of the parasites by additional *Rhipicephalus* tick strains. Additional studies are also needed to address important parameters of vaccine development, such as the duration of protection and the number of vaccination boosters needed for the development of long-term protection. Together with improved tools aimed at controlling tick infestation and the devastating effects of acute babesiosis caused by blood stages of the parasite, transmission-blocking strategies, such as the HAP2-TBV described in this study, could be considered as future cornerstones in the control of bovine babesiosis. However, a control campaign based solely on a TBV approach might pose some risks since animals could be highly susceptible, for instance, to transmission of *Babesia* parasites from ticks derived from wild-type *Babesia* reservoirs^33,34^. The availability of a TBV against *Babesia* parasites would be an important addition to our arsenal to control this parasite and it is highly likely that a fully effective vaccine approach will also require the inclusion of protective blood-stage antigens with transmission-blocking antigens, such as HAP2. Although the results of this study are strongly supportive of HAP2 as a TBV candidate, the experiments were performed in a small number of animals. Work is currently under development to test the effects of HAP2 vaccination in a larger cohort of animals, and to improve a TBV approach based on HAP2. This includes identifying effective blood-stage antigens and testing their performance in a dual blood-stage/TB vaccine that can enhance the effectivity of this vaccination approach towards the control of bovine babesiosis.

We conclude that the results of these transmission experiments strongly support the hypothesis that vaccination of cattle with rHAP2 generates antibody responses that abrogate transmission of *B. bovis* by a competent tick vector. Thus, the data generated in this study fully supports the use of HAP2 as a component of TBV against bovine babesiosis. Because HAP2 is conserved among most, if not all, apicomplexan parasites, the results of this study may also be of relevance for the development of TBVs against other Babesia and Theileria tick-borne parasites, as well as other related arthropod-borne hemoparasites such as Plasmodium, which are responsible for devastating diseases in domestic animals and humans.

## Methods

### Ethics statements

All animal experiments were approved by the Institutional Animal Care and Use Committee, University of Idaho (Protocol IACUC-2016-20). Animals were immediately euthanized upon reaching humane endpoints, determined by fever for more than 3 days, or hematocrit values below 15%. Humane euthanasia was performed by the i.v. administration of sodium pentobarbital at a dose of 10 ml per 100 lbs body weight. Cattle were first anesthetized with Xylazine and brought to a recumbent position prior to euthanasia. Euthanasia was performed by a fully trained technician.

### Expression and purification of recombinant *B. bovis* HAP2 (rHAP2) protein

*B. bovis* HAP2 gene sequence was codon optimized for expression in prokaryotic cells, synthesized, and cloned into vector pET-30a(+) with 6His tag for protein expression in *E. coli* BL21 star (DE3) cells (Fig. 1). After expression, the protein was obtained from inclusion bodies and purified by Ni-column with a purification rate of 95%. The starting volume was 1 L of Terrific medium broth, with a final yield of 18.13 mg. Level of protein purity and expected molecular weight were determined by SDS–PAGE (Fig. 1A) and Western blot (Fig. 1B). Briefly, the proteins were separated in 4–20% Precast TGX gel (BioRad, Hercules, CA, USA) and stained with Coomassie blue using a standard protocol. For Western blot analysis, the SDS–PAGE separated proteins were transferred to a PVDF membrane using manual transfer and probed with HRP-conjugated 6*His, His-Tag Monoclonal antibody (Proteintech, Rosemont, IL, USA). The size marker used in SDS–PAGE and Western blot was Prestained Precision Plus Protein Dual Color Standard (BioRad, Hercules, CA, USA). The original gel and Western blot membrane used in Fig. 1 are shown in Supplementary Notes 1 and 2. Identity confirmation of rHAP2 protein was performed by LC–MS/MS, as per the third-party company’s protocol (GenScript, Piscataway, NJ, USA). The recombinant protein was stored at −80 °C until further use.

### Immunization of calves

An experimental vaccine was formulated by mixing rHAP2 protein at a final concentration of 50 µg/ml with saponin adjuvant (Sigma-Aldrich). This preparation was used for the vaccination of two groups of three calves each calf. This number of experimental animals provides a power of 90%; alpha 0.05^35^ (https://clincalc.com/stats/samplesize.aspx). The two groups of three male Holstein calves (3–4 months of age) were vaccinated subcutaneously with 50 µg of rHAP2 four times, at days 0, 21, 42, and 63 (21-day intervals). Group 1, vaccinated animals (C1646, C1647, and C1649), was immunized with rHAP2 antigen. Group 2, control animals (C1650, C1652, and C1653), received saponin adjuvant only. Pre- and post-inoculation serum samples were collected from each animal and analyzed for the presence of IgG against rHAP2 by iELISA.

### Acquisition of *B. bovis* parasites by *R. microplus* ticks in experimental calves

Eight days after the fourth rHAP2 inoculation, 1 g of *R. microplus* larvae (La Minita strain) were applied under a cloth patch on vaccinated and control calves (day 71 post the first rHAP2 inoculation). Thirteen days after infestation, *R. microplus* nymphs began molting to adults, and calves were inoculated with a frozen stabilate originally containing ~10^7^ *B. bovis* S74-T3Bo Texas strain (virulent, tick-transmissible strain)^10^ iRBC (day 84 post the first rHAP2 inoculation). Serum from all experimental animals was collected before tick application during acute *B. bovis* infection and throughout the experiment to be used in further analysis. All six experimental calves were monitored daily for clinical signs of acute babesiosis, such as fever (>38.8 °C), drop in PCV and parasite load in peripheral blood by qPCR as previously described^36^. During tick infestation and acute *B. bovis* infection, the animals were also monitored for the development of antibodies against rHAP2 by iELISA.

### *R. microplus* feeding on rHAP2 vaccinated animals

Engorged female ticks were collected daily from days 6 to 10 of feeding, coinciding with the peak of *B. bovis* parasitemia. Dropped, engorged female ticks were rinsed in tap water, placed into individual 24-well tissue culture plates, and incubated at 26 °C and 96% relative humidity. After 8 days post-tick collection, 72 replete female ticks per calf were evaluated for the presence of kinetes in their hemolymph for 5 days. A drop of hemolymph was placed on a glass slide, stained with Diff-Quick (Siemens Healthcare Diagnostics), and observed by light microscopy (×100 objective lens). Number of replete ticks dropping per day and total egg masses generated per tick were recorded.

### Transmission of *B. bovis* parasites by *R. microplus*

Larvae from female ticks fed on rHAP2 vaccinated and control calves were used for transmission experiments. Two groups of three males 3–4 months old Holstein calves were used as recipients for tick infestation. Larvae derived from 0.25 g of eggs (~5000 larvae) were applied under a cloth patch to every calf. Considering that the average weight of egg mass generated by individual female ticks recovered after the acquisition feeding in our experiment was 53.65 g (Table 1), we calculated that a sample size of 0.75 g (total of eggs used for generating larvae for the three experimental animals, which represents 1.39% of the estimated population) produced a margin of error of 0.83%, with a confidence level of 95% (home/math/sample size calculator). A group of calves (C99555, C99556, C99557) received a pool of larvae collected from animals previously vaccinated with rHAP2. Another group of control recipient animals (C99562, C99561, C99559) were infested with identical numbers of larvae collected from control non-vaccinated animals. All animals were checked daily for clinical signs of acute bovine babesiosis, such as fever (>38.8 °C), decrease in PCV, and parasitemia by microscopy. Peripheral blood was collected from experimental animals using vacutainer tubes containing EDTA for gDNA extraction before and after tick infestation. Serological analysis was performed on sera obtained from peripheral clotted blood collected from all experimental animals.

### Quantitative PCR and iELISA assays

Genomic DNA was analyzed by qPCR for the detection of the *B. bovis msa-1* gene (:) and serum samples were analyzed for the detection of antibodies against the *B. bovis* HAP2 by iELISA. Indirect ELISA for detection of antibodies against HAP2 was performed by diluting the antigen in 0.05 M carbonate–bicarbonate buffer, pH 9.6, to a final concentration of 0.8 µg/ml. The washes, blocking, and dilution of the antibodies were performed using the same buffer: 0.2% I-block in phosphate buffer saline (PBS) with 0.1% Tween 20, PBST. Briefly, fifty µl of the diluted antigen was added per well in 96-well Immulon^®^ 2 HB Flat Bottom MicroTiter^®^ ELISA plates (Thermo Scientific) and incubated overnight at 4 °C. After incubation, ELISA plates were washed three times with 200 µl per well of buffer and incubated with 300 µl per well of blocking buffer for 1 h at 30 °C. Then, bovine serum was diluted 1:400, and 50 µl of diluted serum was added per well. Plates were incubated for 1 h at 30 °C. The diluted serum was discarded and after five washes, diluted anti-bovine HRP conjugated (1:1100) was added per well. Plates were incubated for 45 min at 30 °C and washed four times with washing buffer, followed by two washes with PBST. The colorimetric reaction was developed by adding 55 µl per well of 1-Step Ultra TMB-ELISA substrate solution (Thermo Scientific) for 10 min. The reaction was stopped by adding 55 µl per well of 2 M H_2_SO_4_ solution and the absorbance was read in each well at 450 nm using a spectrophotometer.

An iELISA for the detection of antibodies against RAP-1/CT was performed by diluting rRAP-1/CT to a final concentration of 5 µg/ml and experimental sera diluted 1:50. The remaining protocol was performed as specified above. Serum from calf C37816 was derived from an experimentally *B. bovis*-infected cattle^17^ and used as control. The monoclonal antibody BABB75^37^ was used as a control in the iELISA. Pre-immune and immune sera from calves C1707, C1705, C1703, C1706, C11325, C1738, C1736, and C1735 used in the Supplementary Fig. 1 were generated in a previous study^10^.

A real-time qPCR to detect *B. bovis* gDNA was performed^36^. Briefly, gDNA was extracted from bovine peripheral blood using QIAmp DNA Blood Mini Kit (QIAGen) and 100 ng was used for amplification. Primers (5′-GATGCGTTTGCACATGCTAAG-3′ and 5′- CGGGTACTTCGGTGCTCTCA-3′) and probe (5′-CACGCTCAAGTAGGAAATTTTGTTAAACCTGGA-3′) specific for *B. bovis msa-1* were used at 60 °C of annealing temperature. Reactions were performed in duplicates in 20 µl using 200 nM of primes and 100 nM of probe. Quantification of *B. bovis msa-1* copies was calculated based on a plasmid standard curve. Efficiency of amplification was performed to evaluate the analytical sensitivity of the qPCR and amplicons were sequenced to assess the specificity of the reaction. Results are presented as copies of *B. bovis msa-1* per 0.5 µg of gDNA. The parasite load data was analyzed for statistical significance using the Mann–Whitney test (non-parametric test).

### Statistical analysis

Tick-derived parameters were analyzed with a mixed model with treatment as a fixed effect, calf within treatment as random, and age of calf as a covariant. Calf-derived measurements were analyzed with a mixed model with treatment as a fixed effect, sire as a random effect, and age of the calf as a covariant. Collecting at least 14 ticks per animal per day for analysis provided 90% statistical power. Statistical analysis of changes in PCV and temperature between experimental animals was performed by Student’s *t*-test and Fisher test and ANOVA using GraphPad Prism, GraphPad, USA.

### Reporting summary

Further information on research design is available in the Nature Research Reporting Summary linked to this article.

### Supplementary information

Supplementary Information

reporting summary

## Data Availability

All data associated with this study are present in the paper. Requests for resources, data, and reagents should be directed to the lead contact, C.E.S. (carlos.suarez@usda.gov). All unique reagents described in this study are available upon request to the lead author with a completed Materials Transfer Agreement.
