# Supplementary material for: Vaccination of cattle with the Babesia bovis sexual-stage protein HAP2 abrogates parasite transmission by Rhipicephalus microplus ticks
Source: NPJ Vaccines. 2023 Sep 27;8:140. doi: 10.1038/s41541-023-00741-8 (PMC10533483; doi:10.1038/s41541-023-00741-8)

A

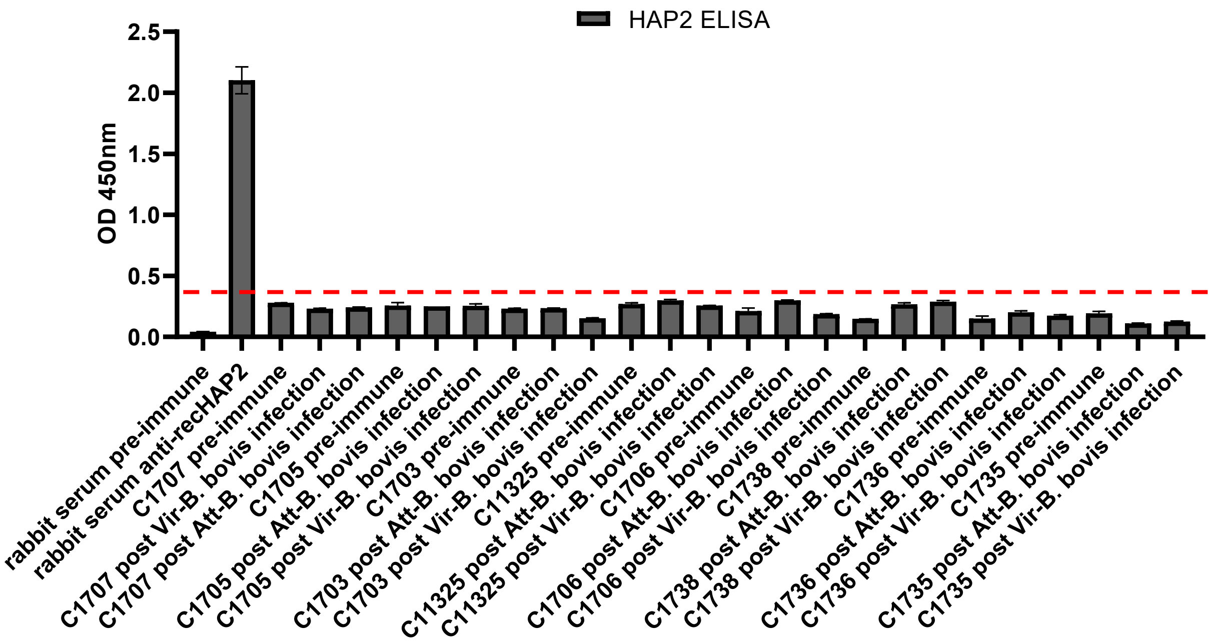

B

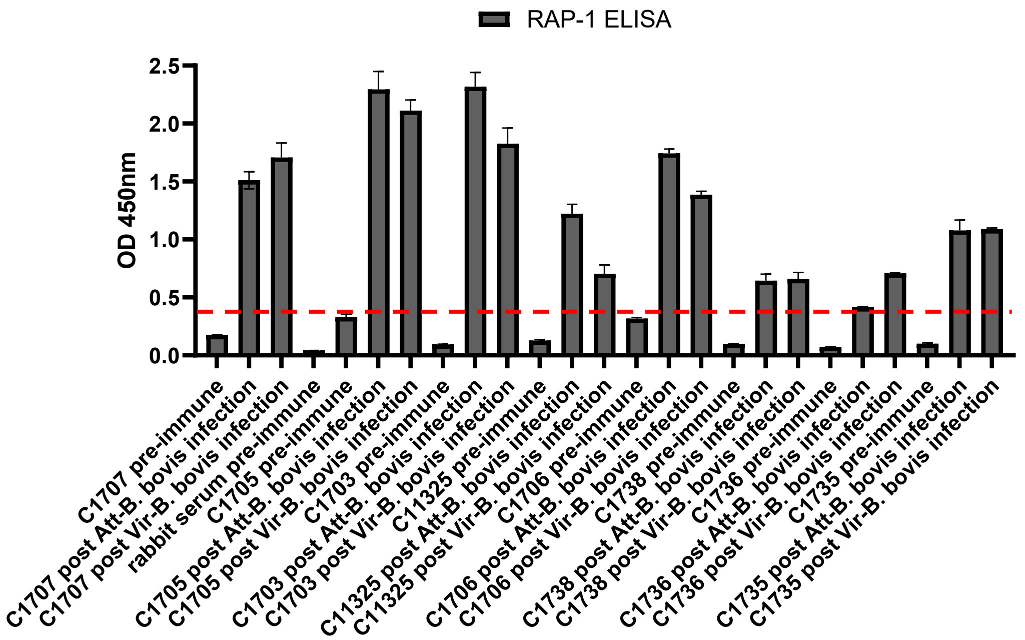

Supplementary Figure 1. Serum samples from eight cattle were evaluated for the presence of antibodies to HAP2 (panel A) and RAP-1 (panel B) before infection (pre-immune), post-infection with an attenuated (Att) *B. bovis* strain, and post-infection with a virulent (Vir) *B. bovis* strain. A hyperimmune rabbit serum against recombinant HAP2 (recHAP2) was used as a positive control in the HAP2 ELISA. The red dashed line represents the cutoff value of the iELISA, calculated as the OD of non-infected cattle sera +3SD)**.**

Supplementary Note 1: Original Western blot using anti HIS antibodies used in Fig 1a: Lanes 1-8: rHAP; Lane 10: HIS-rec protein control; SM; Size Markers

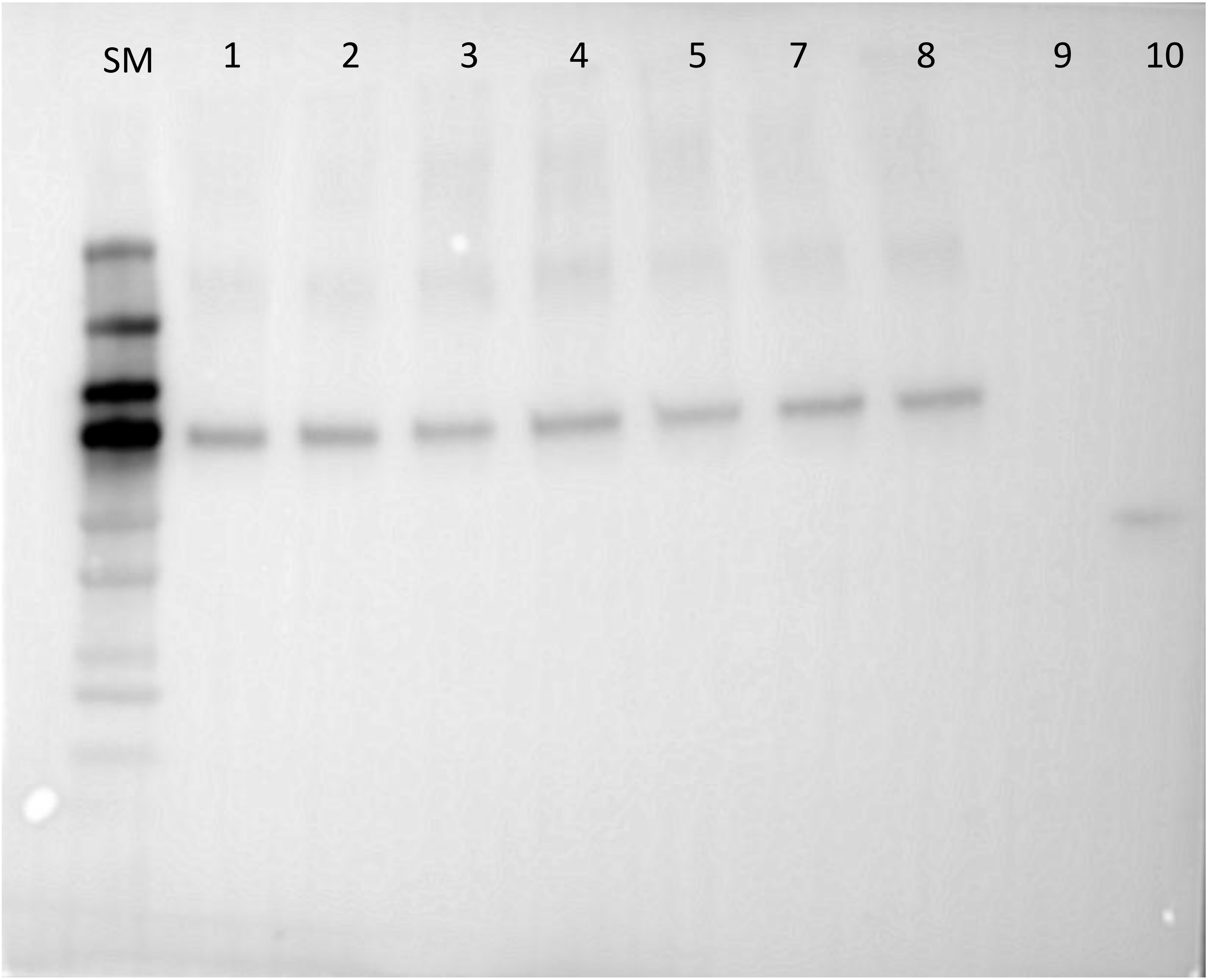

Supplementary Note 2: Original Coomassie stained gel. Lanes 1-7: rHAP2; Lane 8: Control rec protein; SM: Size Markers
SM and lane 1 were used for Figure 1a

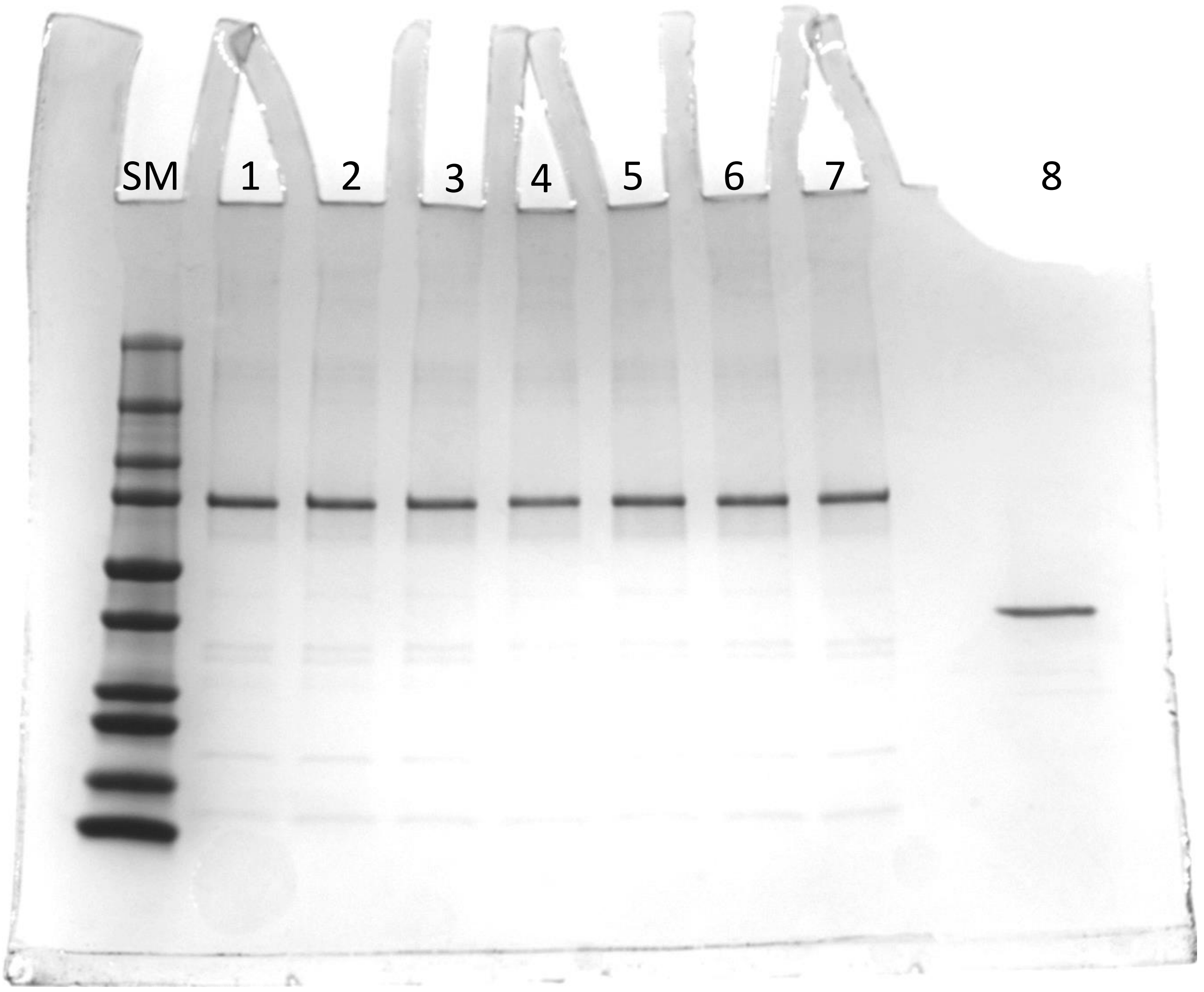

Supplement: Supplementary file 1 — Supplementary Information [file 41541_2023_741_MOESM1_ESM.pdf]
